# Widespread presentation of brown tumors mimicking multiple myeloma

**DOI:** 10.1186/s41824-023-00168-3

**Published:** 2023-06-08

**Authors:** Julien Duyck, Katrien Spincemaille, Jan Verfaillie, Caressa Meert, Kristoff Muylle

**Affiliations:** 1 Department of Nuclear Medicine, AZ Delta, Roeselare, Belgium; 2 Department of Endocrinology, AZ Delta, Roeselare, Belgium; 3 Department of Otolaryngology, AZ Delta, Roeselare, Belgium; 4 Department of Hematology, AZ Delta, Roeselare, Belgium; 5 Beselare, Belgium

**Keywords:** Brown tumors, Osteitis fibrosa cystica, Primary parahyperthyroidism, FDG-PET/CT, Bone SPECT/CT, ^99m^Tc-sestamibi scintigraphy

## Abstract

Brown tumors or osteitis fibrosa cystica has become a rare presentation of primary hyperparathyroidism in up-to-date clinical practice. Here, we describe a case of longstanding untreated hyperparathyroidism presenting itself with brown tumors in a 65-year-old patient. During the diagnostic work-up of this patient, bone SPECT/CT and ^18^F-FDG-PET/CT revealed multiple widespread osteolytic lesions. Differentiating from other bone tumors such as multiple myeloma is challenging. In this case, the final diagnosis was made by integrating the medical history, biochemical diagnosis of primary hyperparathyroidism, pathological findings and medical imaging.

## Main text

A 65-year-old female patient was referred to the orthopedics department with complaints of pain in both legs.

**Fig. 1:**
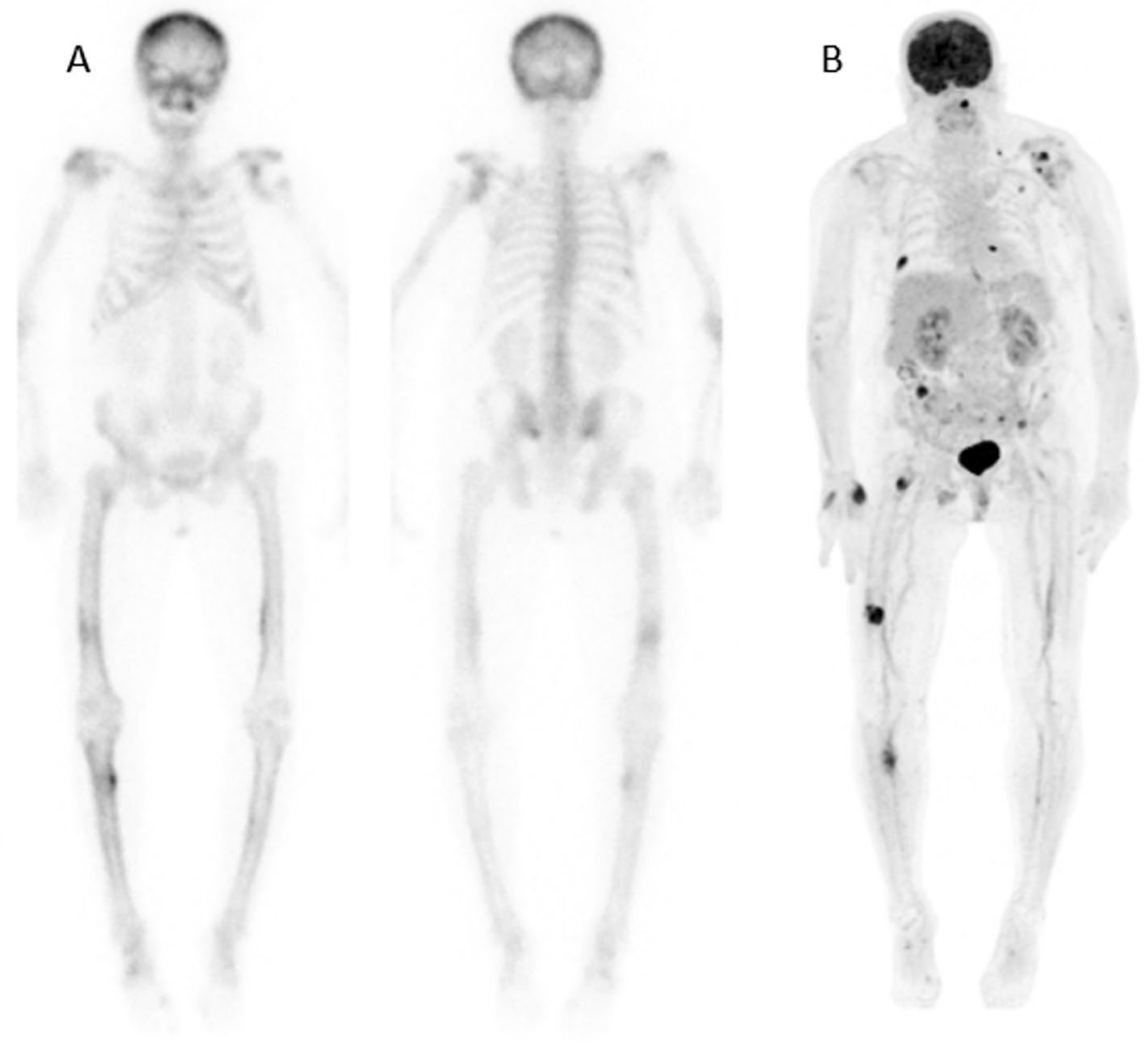
Whole body bone scintigraphy (anterior and posterior view; **A**) and ^18^F-FDG-PET/CT (maximum intensity projection; **B**)

After clinical examination, a bone scintigraphy with SPECT-CT (Figs. 1A/2A) was performed, revealing multiple osteolytic lesions with associated, variable (predominantly moderate) osteoblastic activity in the axial and peripheral skeleton affecting the skull (left maxillary sinus), the left scapula and bilateral lesions in the ribs, pelvis, femur and tibia. The focal higher osteoblastic activity in the right tibia might be related to an associated microfracture. A blood sample showed hypercalcemia (3.86 mmol/L; normal range 2.20–2.55) and increased alkaline phosphatase (240 U/L; normal range: 35–105) with normal blood cell counts, a preserved renal function and the absence of monoclonal antibodies in serum.

**Fig. 2:**
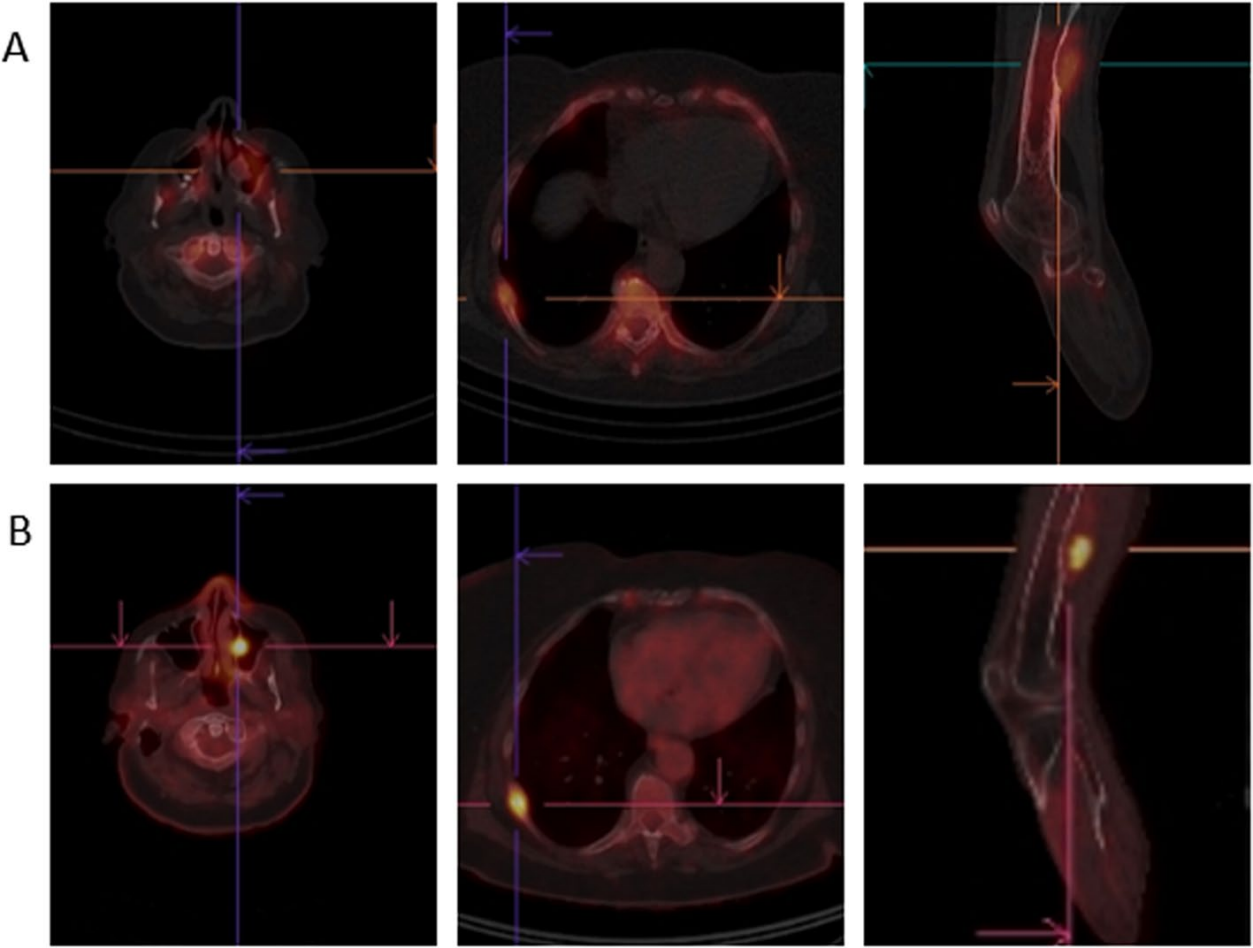
Comparison of the presentation of 3 bone lesions on bone SPECT/CT (upper row; **A**) and ^18^F-FDG-PET/CT (lower row; **B**), respectively, in the skull (left maxillary sinus), a rib and the right femur

Further work-up with ^18^F-FDG-PET/CT (Figs. 1B/2B) confirmed the presence of multiple osteolytic lesions, all hypermetabolic and some with cortical breakthrough and expansile growth, compatible with osteolytic bone metastases or a non-secretory myeloma. Compared to bone SPECT/CT, ^18^F-FDG-PET/CT was more sensitive for depicting small bone lesions (e.g. in ribs) and allowed a better discrimination of disease related lesions from reactive bone remodeling, for example, nearby the left shoulder prothesis.

A CT-guided biopsy of an osteolytic lesion in the right tibia showed tumoral tissue rich in osteoclast type giant cells. This finding in combination with hypercalcemia and hyperparathyroidism in a new blood test (PTH: 1121 ng/L; normal range: 15–65) as well as the imaging results lead to a diagnosis of so-called brown tumors.

Review of the patients’ medical history revealed that the patient had been referred to an endocrinologist 14 years earlier (in 2008) because of hypercalcemia found in a routine blood test. Since PTH was elevated (231 pg/ml, normal 15–65 pg/ml) and hypocalciuric hypercalcemia was excluded, primary hyperparathyroidism was suspected. A supra-centimetric parathyroid adenoma posterior from the left thyroid lobe was identified by imaging with a planar ^99m^Tc-sestamibi scintigraphy and echography. Although the patient was referred to the surgery department for a parathyroidectomy, the patient was lost to follow-up and the surgery never took place.

**Fig. 3:**
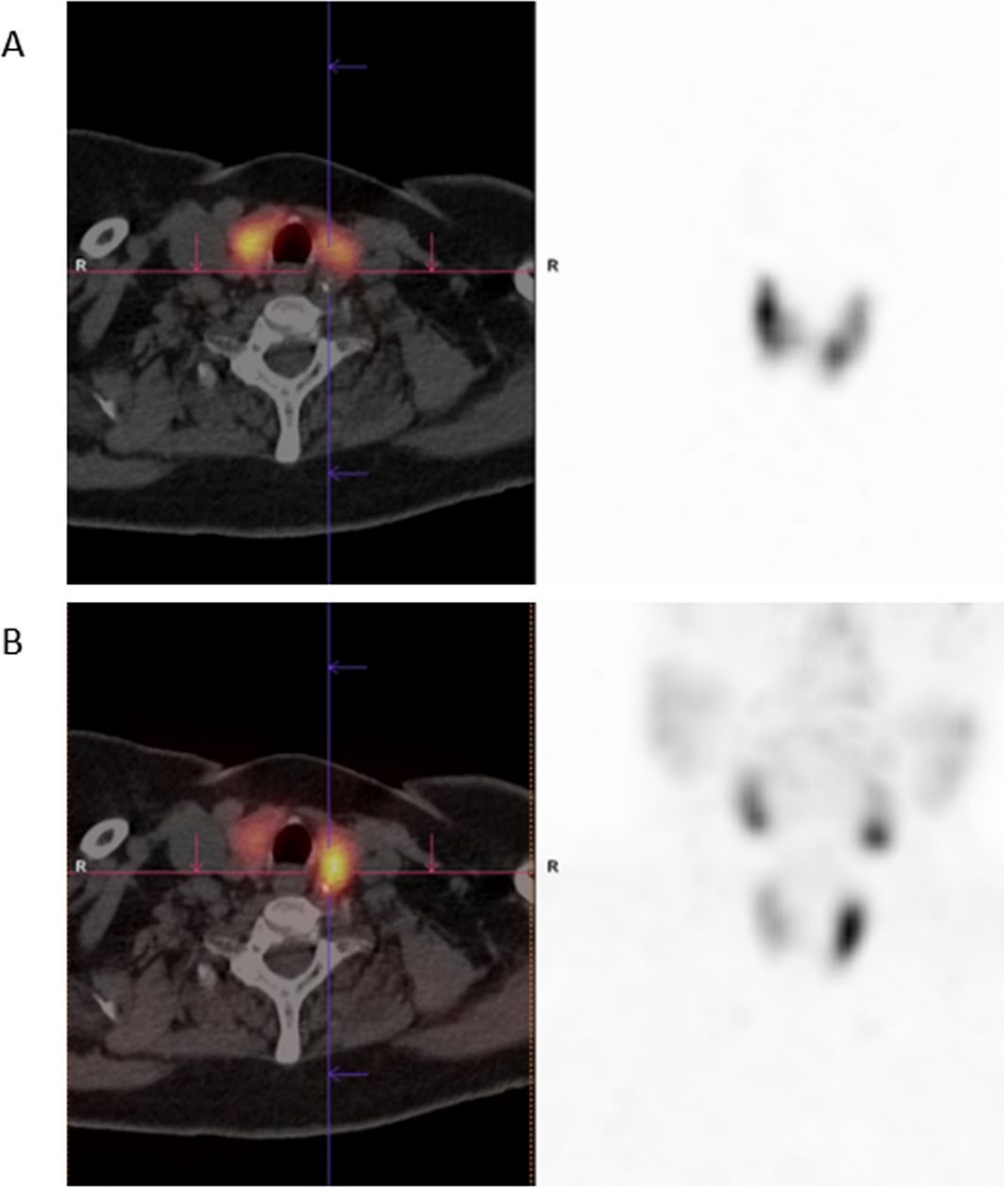
A dual isotope ^123^I/^99m^Tc-sestamibi SPECT/CT confirmed the presence of a ^99m^Tc-sestamibi-avid nodule (lower row; **B**, without ^123^I-uptake upper row; **A**) posterior from the left thyroid lobe

A dual isotope ^123^I/^99m^Tc-sestamibi SPECT/CT (Fig. 3) and echography was repeated, confirming the presence of a parathyroid adenoma posterior from the left thyroid lobe. After surgical resection of the parathyroid adenoma (dimensions: 24 × 18 mm), PTH and calcium in serum normalized. Given the osteoporosis (confirmed by a bone density scan; *T*-score: − 3.4 at the proximal femur) secondary to the longstanding hyperparathyroidism, a treatment with biphosphonates (zoledronic acid 4 mg) is administered every 4 weeks.

## Conclusion

Brown tumors are degenerative cystic formations caused by persistent hyperparathyroidism. These usually are the end stage lesions of constantly elevated parathyroid hormone (PTH) (Chew and Huang-Hellinger 1993; Vanitcharoenkul et al. 2021; Bilezikian et al. 2005; Silverberg and Bilezikian 1996). These have become increasingly rare to find because of the early detection of hyperparathyroidism due to routine biochemical screening (Vanitcharoenkul et al. 2021; Bilezikian et al. 2005; Silverberg and Bilezikian 1996; Pappu et al. 2016; Liu et al. 2013). Radiologically these tumors present as lytic lesions or multilobular cystic changes of the bone (Misiorowski et al. 2017). The differential diagnosis with other bone tumors such as osteolytic bone metastasis, multiple myeloma, bone cysts and giant-cell tumor can be challenging by medical imaging alone (Van der Woude and Smithuis 2010; Kalathas et al. 2010). Although nowadays it has become a rare imaging finding, osteitis fibrosa cystica should be considered in the differential diagnosis in patients presenting osteolytic lesions particularly with cortical breakthrough, expansile growth and hypermetabolic features on ^18^F-FDG-PET/CT. Incorporation of pathological findings and biochemical analysis—showing high PTH, high calcium and low phosphate—is needed to diagnose brown tumors in the context of longstanding hyperparathyroidism (Misiorowski et al. 2017; Van der Woude and Smithuis 2010).

## Data Availability

No data or material was interpreted for this case report.
